# Temporal Changes in Continuous Glucose Monitoring Profiles in Health Care Workers Participating in a Digitally Delivered Metabolic Wellness Program

**DOI:** 10.1177/19322968261463562

**Published:** 2026-07-13

**Authors:** Bariq S. Firmansyah, Ronesh Sinha, Ashutosh Sabharwal, Cheryl D. Stults, Joseph Wilcox, Katie Gillespie, Rekha Viswanathan, Richard Chapman, Susan McClendon, David Kerr

**Affiliations:** 1 Department of Electrical and Computer Engineering, Rice University, Houston, TX, USA; 2 Sutter Health, Palo Alto, CA, USA; 3 Center for Health Systems Research, Sutter Health, Palo Alto, CA, USA; 4 Center for Health Systems Research, Sutter Health, Walnut Creek, CA, USA; 5 Sutter Health, Sacramento, CA, USA; 6 Center for Health Systems Research, Sutter Health, Santa Barbara, CA, USA

**Keywords:** digital health, metabolic wellness, continuous glucose monitor, mobile health, lifestyle intervention, educational programs, behavioral changes

## Abstract

**Background:**

Health care workers face elevated cardiometabolic risks due to irregular hours and chronic stress, yet targeted interventions are limited. This study evaluated an 8-week digital Metabolic Wellness Program (MWP), combining lifestyle education with real-time continuous glucose monitoring (CGM).

**Methods:**

One hundred health care workers and dependents within a California health care system completed the MWP and were stratified by baseline time in tight range (TITR) 70 to 140 mg/dL. Continuous glucose monitoring (CGM)-derived metrics (average glucose [AG], glycemia risk index [GRI], time in range [TIR] 70-180 mg/dL, TITR, coefficient of variation [CV]) were evaluated via static pre-post comparison. These were supplemented by novel temporal change metrics: mean change index (MCI, average daily change relative to baseline) and improvement consistency index (ICI, % days with improvement). Engagement and behavioral changes were also assessed.

**Results:**

Static pre-post comparisons of CGM-derived metrics showed no significant glycemic changes cohort-wide or within TITR strata. However, temporal change metrics revealed improvements across participants. The low TITR (<70%) Group showed comprehensive improvements: AG (MCI −17.2 mg/dL), GRI (MCI −15.3), CV (MCI −3.2%), TIR (MCI 8.5%), and TITR (MCI 8.8%), with consistent improvements in GRI (ICI 72.1%), TIR (ICI 70.5%), and CV (ICI 73.3%), all *P* < .05. The high TITR (≥90%) group improved CV (MCI −1.6%, ICI 69.5%, *P* < .001). Metabolic Wellness Program engagement was associated with positive behavioral changes (*P* < .001), which were correlated with improved temporal change metrics (eg, TIR MCI: *r* = 0.31, *P* < .05).

**Conclusions:**

While static CGM metrics remained unchanged, MWP participation was associated with significant behavioral and temporal glycemic improvements, particularly among those with lower baseline TITR. Long-term sustainability warrants further investigation.

## Introduction

Continuous glucose monitoring (CGM) provides real-time physiological data that can facilitate positive behavior change, promoting evidence-based nutrition recommendations,^1^ and supporting guidance for physical activity.^2^ While CGM is a standard of care for non–insulin-treated type 2 diabetes (T2D),^3^ recent over-the-counter availability has expanded its use to individuals without T2D. However, the widely used time-in-range (TIR) metric between 70 and 180 mg/dL lacks utility for individuals without diabetes, who are almost always (>95%) within this range.^4^ More recently, discussions within the diabetes community suggest technology advances may allow tighter glycemic targets with a move toward a “time in tight range” (TITR) between 70 and 140 mg/dL in type 1 diabetes (T1D).^5^ Beyond insulin-treated diabetes, evidence suggests additional glucose metrics can differentiate established T2D from prediabetes or at-risk individuals.^6^ Combining CGM metrics with other wearable data offers novel insights into relationships between glucose profiles, food choices, and physical activity that may become therapeutic targets.^7-9^

Health care workers face elevated cardiometabolic risks due to prolonged or irregular working hours, chronic psychosocial stress, and shift work, which can disrupt circadian rhythms and metabolic homeostasis.^10-12^ This may be reflected in dynamic glucose changes over short periods. While standard summary CGM metrics are based on average glycemic changes over the duration of sensor wear, we hypothesize that additional metrics may provide information on shorter-duration fluctuations reflecting dynamic glycemic changes.

Traditional methods assess glycemic changes by comparing CGM-derived metrics at baseline and endpoint. However, during education programs, static pre-post comparison may not adequately capture real-time glycemic changes reflecting participant engagement. To address this limitation, we propose two novel temporal change metrics: the mean change index (MCI), which quantifies the average daily deviation from baseline, and the improvement consistency index (ICI), which quantifies the percentage of days with improvement compared with baseline (eg, days with lower average glucose [AG] or higher TITR). We applied temporal change metrics alongside static CGM metrics to evaluate a digitally delivered Metabolic Wellness Program (MWP) combining education with CGM, targeting health care workers and their dependents within an integrated health care system in California. We further examined the associations between MWP engagement, self-reported behavioral changes, and glycemic changes.

## Methods

### Participants

The study (NCT06790472) was approved by the Sutter Health Institutional Review Board. Participants were employees and dependents of Sutter Health, an integrated health care network in California. Inclusion criteria included self-reported: (1) non–insulin-treated T2D, (2) prediabetes, or (3) no T2D/prediabetes diagnosis (metabolically healthy). Quotas intentionally oversampled those with the highest potential glycemic benefit: 50% non–insulin-treated T2D, 30% prediabetes, and 20% healthy. Exclusion criteria were insulin use, pregnancy, or chronic kidney disease on dialysis. Recruitment via corporate email to approximately 50 000 employees yielded 1858 completed screening forms, from which participants were selected using quota-based sequential enrollment. All study aspects were executed digitally.

### Metabolic Wellness Program

**Figure 1.**
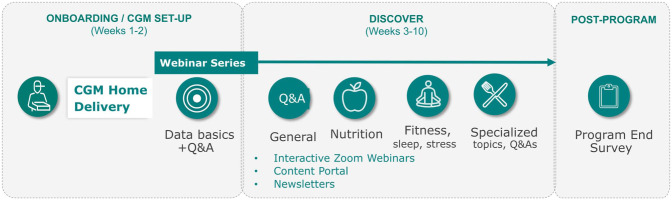
The MWP study consisted of three phases: a 2-week onboarding phase, during which participants received CGM devices and learned the basics of CGM data, an 8-week discovery phase (the MWP program) featuring weekly themed educational webinars, and a post-program phase, where participants completed the program end survey.

The MWP is an established, digitally delivered program offered through Sutter Health. The study spans three chronological phases: a 2-week onboarding phase, an 8-week discovery phase (the core MWP program), and a post-program phase (Figure 1).^13^ During onboarding, participants received Abbott Freestyle Libre 3 CGM devices (Abbott Diabetes Care, Alameda, California) via the corporate pharmacy system. These devices measure interstitial fluid glucose every minute and store the data every 5 minutes. Participants were asked to upload these data to the Sutter Health server, where they were de-identified before analysis. The discovery phase featured weekly webinars led by a physician and a registered dietitian covering nutrition, physical activity, sleep, stress management, and CGM utility. Participants accessed live webinars or recordings via a web portal, which also hosted supplemental educational videos and documents. Weekly newsletters were emailed to summarize key content. Post-program, participants completed surveys regarding program engagement, behavioral changes, and self-reported weight.

### Temporal Change Metrics

Five CGM-derived metrics were assessed: AG in mg/dL, glycemia risk index (GRI), coefficient of variation (CV), TIR 70 to 180 mg/dL, TITR 70 to 140 mg/dL.^14-16^ These CGM metrics can be calculated for any time period, such as baseline, endpoint, or daily throughout an intervention.

**Figure 2.**
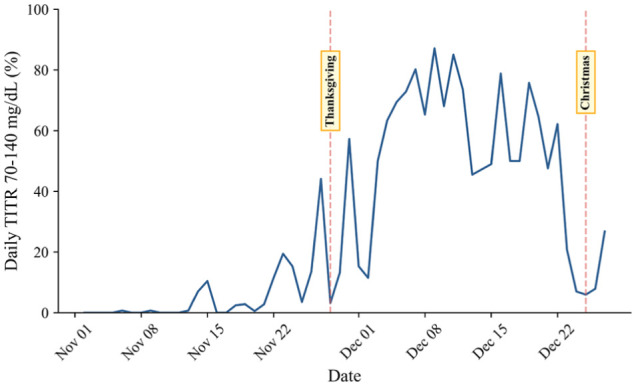
Daily TITR throughout the program of a selected participant and covering the Thanksgiving and Christmas holiday periods.

Comparing CGM metrics only at baseline and endpoint may fail to capture dynamic glycemic changes during a longitudinal lifestyle intervention like MWP. For example, a participant’s daily TITR might improve rapidly during the intervention but attenuate during the final days of the program, which coincided with the Christmas holiday period (Figure 2). Baseline versus endpoint CGM-derived metrics would classify this participant as having limited success, obscuring multiple days of mid-program improvement (here, achieving a higher daily TITR than baseline). To capture these intra-intervention dynamics, we propose two novel temporal change metrics: MCI and ICI.

#### Mean Change Index

The MCI quantifies the average daily change in a CGM metric relative to baseline:

$$\mathrm{MCI}=\frac{\Sigma_{d=b+1}^{D}m(d)-m(\mathrm{baseline})}{D-b}$$

where D is the total CGM wear duration (days) and b is the baseline duration (days). Thus, D−b in the denominator represents the number of intervention (non-baseline) days. m(d) is the CGM metric value on day d, and m(baseline) is the baseline value of that CGM metric. The CGM metric *m* can be any CGM metric, such as AG, GRI, TIR, TITR, or CV. For metrics where higher values indicate better control (TITR, TIR), MCI>0 represents improvement. In contrast, for metrics where higher values are less desirable (AG, GRI, CV), MCI<0 represents improvement. For example, an AG MCI of −20 mg/dL indicates that the daily glucose was, on average, 20 mg/dL lower than baseline, while a TITR MCI of +10% indicates that an individual spent an additional 2.4 hours (10% of 24 hours) per day within the 70-140 mg/dL range compared with baseline.

#### Improvement Consistency Index

$$\mathrm{ICI}=\frac{100\%\;*\;(\mathrm{Number}\;\mathrm{of}\;\mathrm{improved}\;\mathrm{days})}{D-b}$$

While the MCI quantifies the average magnitude of change, the ICI quantifies the consistency of improvement by calculating the percentage of intervention days that outperformed baseline. Specifically, an improved day is defined as a day when the metric value is higher than baseline for metrics where higher values indicate better control (TITR, TIR), or lower than baseline for metrics where higher values are less desirable (AG, GRI, CV). An ICI >50% indicates that the participant maintained improvement over the majority of the intervention days.

### Study Outcomes

The primary outcome was change in TITR. In addition, changes in four other CGM metrics were assessed: AG, GRI, TIR, and CV, using (1) static pre-post comparisons (endpoint minus baseline values) and (2) temporal change metrics (MCI and ICI) as described above. Additional secondary outcomes included self-reported program and CGM engagement, behavioral changes, and weight changes.

### Baseline and Endpoint Definitions

The baseline period was defined as the first 3 days of CGM wear. Although a 14-day baseline would be ideal for capturing more stable CGM metrics, the use of unblinded CGM in the study meant that a longer duration would capture early intervention responses rather than a true physiological baseline. The endpoint period was defined as the final 14 days of CGM wear.

### Baseline Glycemic Status

Prior research suggests that individuals without T2D have a median TITR of 96% (interquartile range [IQR]: 93%-98%).^17^ Elsewhere, a target for TITR has been suggested as ≥70%.^14^ Based on these studies, participants were stratified by baseline TITR into three groups:

1. High TITR group: TITR ≥ 90%
2. Moderate TITR group: 70% ≤ TITR < 90%
3. Low TITR group: TITR < 70%

### Survey/Self-Reported Data

#### Program Engagement Score (0-20)

A composite score derived from five items (each rated 0 = not engaged, 4 = very engaged) evaluating engagements with four program elements (webinars, educational videos, newsletter, and web portal), and the overall program engagement.

#### CGM Engagement Score (0-24)

A composite score derived from six items (each rated 0 = not engaged, 4 = very engaged) assessing the engagement with CGM to inform lifestyle choices (food, exercise, sleep, and stress), overall CGM engagement, and the desire for continued use of CGM after the program.

#### Behavior Change Score (−6 to +6)

A composite score derived from six items (each rated −1 = negative change, 0 = no change, +1 = positive change) assessing changes in six behavioral aspects at program end relative to baseline: portion sizes, carbohydrate intake, healthy food choices, sugar intake, eating out frequency, and physical activity.

#### Self-Reported Weight

Participants self-reported their weight (lbs) at the start and end of the program.

### Statistical Analyses

Analyses were performed in Python (v3.11.6) using SciPy (v1.11.4) and scikit-posthocs (v0.11.2). Normality was evaluated using the Shapiro-Wilk test. Two-tailed, sign-flip permutation tests were used instead of one-sample *t* tests to examine whether mean changes significantly differed from zero (or from 50% for ICI), because most continuous variables (MCI, ICI, static CGM metrics, and self-reported weight) were non-normally distributed within the overall cohort and within each baseline TITR group. The Kruskal-Wallis test, followed by Dunn’s post-hoc test with a Bonferroni correction, was used for between-group comparisons of behavior change scores, as the score is an ordinal composite measure derived from a Likert scale. Spearman’s rank correlation evaluated relationships between the behavior change score and temporal change metrics, as it accommodates ordinal data and non-normal distributions using ranks. Unless specified otherwise, data are expressed as Mean (95% confidence interval). Statistical significance was set at α=0.05 .

## Results

A total of 100 participants aged 21 to 71 years enrolled; 84 opted for CGM (average wear: 42.6 days). Of the 84 participants, 75 wore the CGM for at least 28 days and were stratified by baseline TITR (Table 1). Program adherence and data completeness are detailed in Table 2.

**Table 1.** Baseline Participant Characteristic.

Characteristics	Mean ±SD or percentage
Total participants at enrollment	100
Self-reported diagnosis	No diagnosis (20%), prediabetes (30%), T2D (50%)
Sex	Male (22%), female (68%), unreported (10%)
Race/ethnicity	
White non-Hispanic	46%
Asian	22%
Hispanic	16%
Black	3%
Other/unreported	13%
Age	49.6 ± 11.1 years
Baseline HbA1c^a^	6.44 ± 1.63 % or 46.93 ± 17.83 mmol/mol
Baseline TITR groups^b^	
High TITR	46.7%
Moderate TITR	30.7%
Low TITR	22.6%

a HbA1c available within three months prior to the program start date, n = 41 from the electronic health record.

b Of the 75 participants who adhered to CGM (defined as having at least 28 days of CGM data, with at least 8 hours of data each day).

**Table 2.** Program Adherence and Data Completeness.

Metrics	Mean ±SD or percentage
Withdrew from the study	1%
CGM adherence	
CGM wear duration	42.6 ± 11.5 days
Uploaded CGM data	84%
Adhered to wearing CGM^a^	75%
Survey completeness	
Completed baseline survey	94%
Completed final survey	71%
Completed behavior changes questions	68%
Completed behavior changes and engagements questions	66%
Completed behavior and engagement questions and adhered to CGM	58%
Clinical data completeness	
Had paired baseline and post-program weight measurements	65%
Had baseline, post-program, and paired HbA1c measurements	41% (baseline HbA1c), 49% (post-program HbA1c), 25% (Paired HbA1c)

a Defined as having at least 28 days of CGM data, with at least 8 hours of data each day.

### Static (Pre/Post) CGM-Derived Metrics

There were no statistically significant changes in any CGM metric (AG, GRI, TIR, TITR, CV) when comparing baseline to endpoint, either overall or when stratified by baseline TITR. A Significant AG improvement (−4.5 mg/dL [−8.4 to −0.5], *P* = .04) was observed only in self-reported metabolically healthy participants.

### Mean Change Index

**Figure 3.**
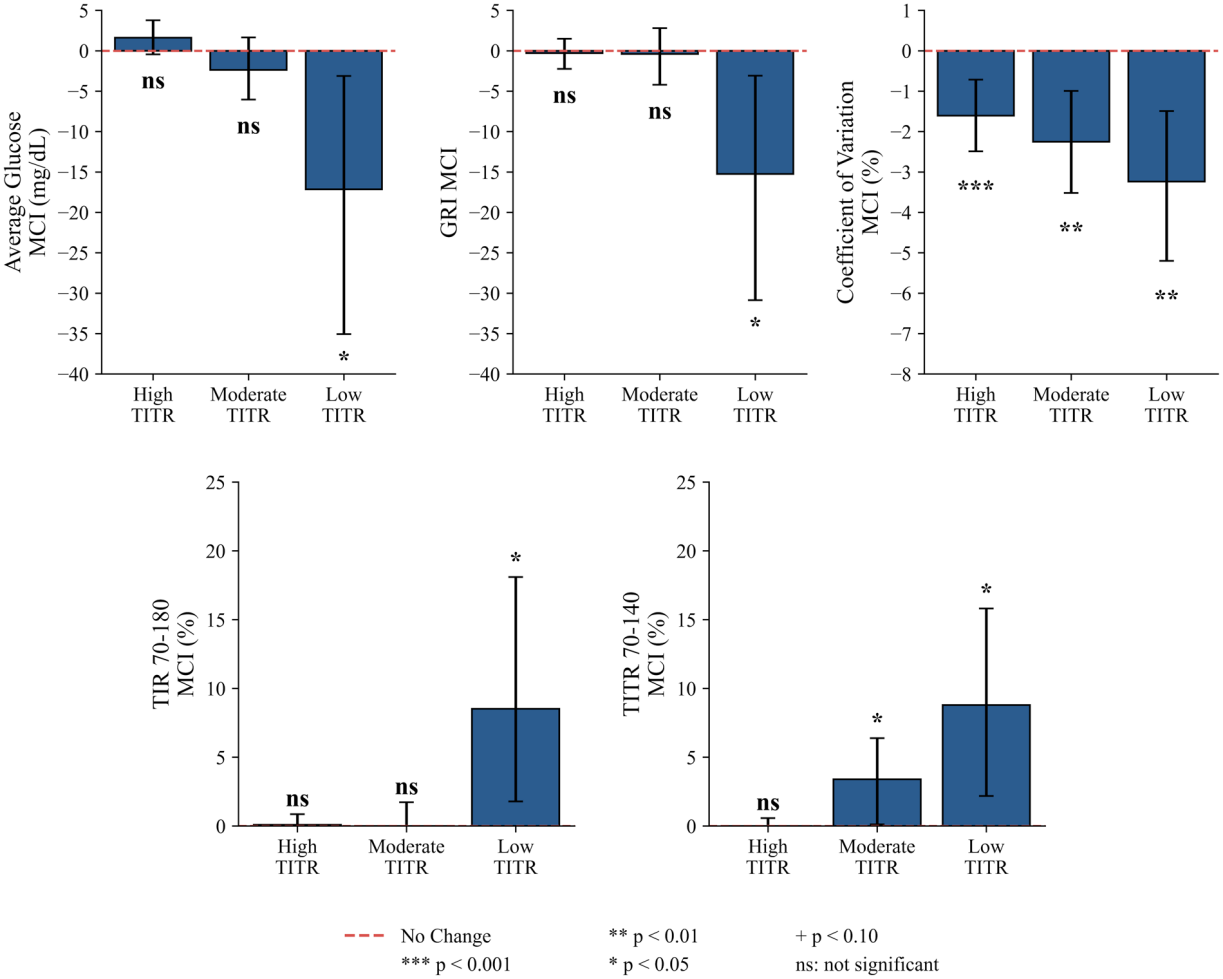
Mean change index (MCI) of each baseline TITR group (baseline was computed over the first 3 days of CGM wear) in five key metrics: average glucose (AG), glycemia risk index (GRI), coefficient of variation (CV), time-in-tight-range (TITR), and time-in-range (TIR). Positive MCI values indicate improvement for TIR and TITR, while negative MCI values indicate improvement for AG, GRI, and CV. Data shown as mean and 95% CI. The *P* value for each TITR group indicates that the MCI value in that subgroup differs significantly from zero, evaluated using a two-tailed, sign-flip permutation test.

The MCI was evaluated independently within each baseline TITR group to determine whether the group’s mean values differed significantly from zero (Figure 3). The low TITR Group showed improvements across all metrics: AG (−17.2 mg/dL [−35.4 to −3.2]), GRI (−15.3 [−31.6 to −3]), CV (−3.2% [−5.1 to −1.4]), TIR (8.5% [1.7-17.9]), and TITR (8.8% [2.1-15.6]), all *P* < .05. Significant improvements in the moderate TITR group were observed for CV (–2.3% [−3.5 to −1.0]), and TITR (3.4% [0.1-6.4]), both *P* < .05. The high TITR group maintained high TITR levels while achieving significant CV reductions (−1.6% [−2.5 to −0.7], *P* < .01). In a secondary analysis stratified by self-reported baseline diagnosis, T2D individuals showed improvements across all metrics except AG, whereas prediabetes and metabolically healthy individuals showed improvements in CV (Supplementary Figure 1).

### Improvement Consistency Index Results

**Figure 4.**
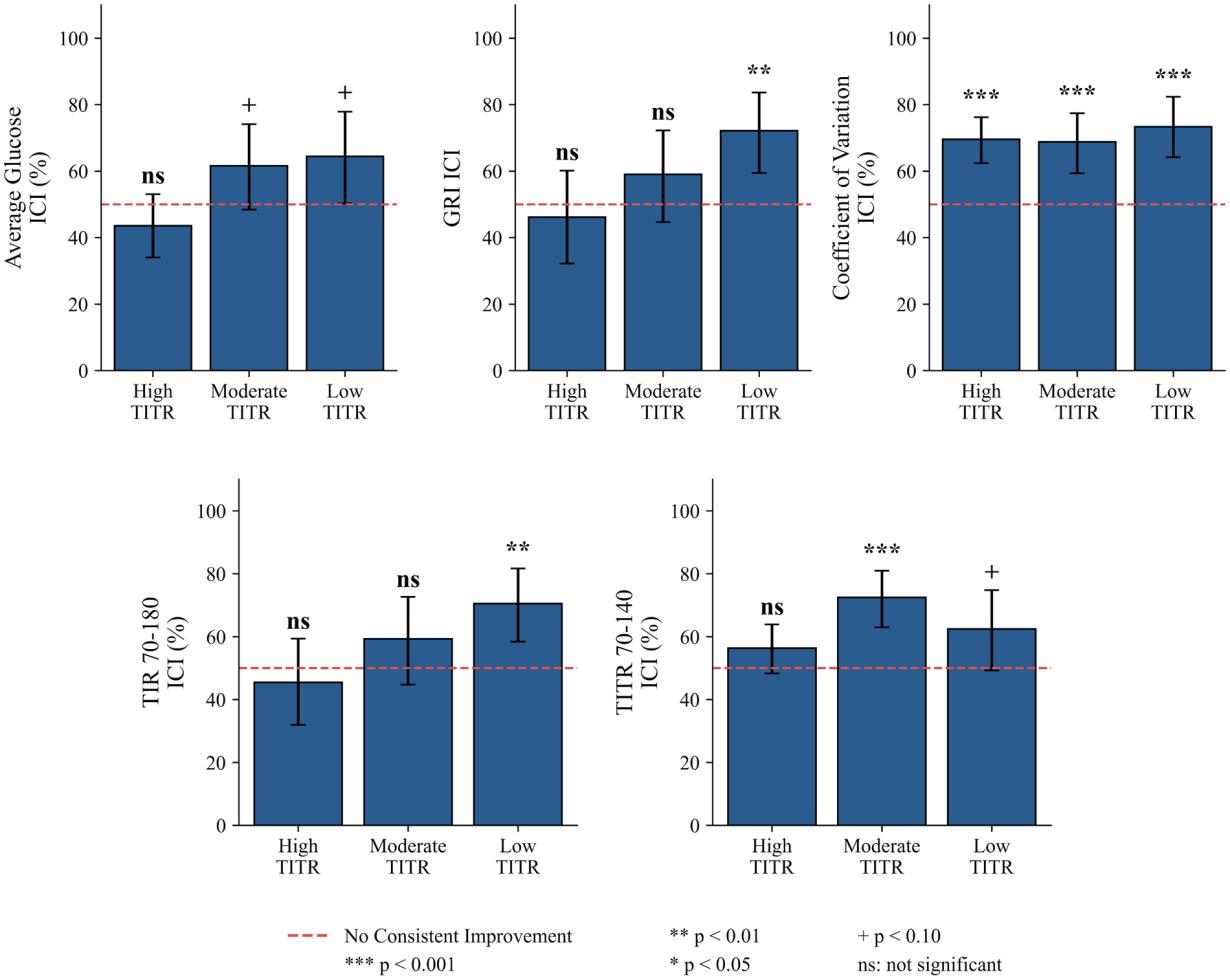
Improvement consistency index (ICI) of each baseline TITR group (baseline was computed over the first 3 days of CGM wear) in five key metrics: average glucose (AG), glycemia risk index (GRI), coefficient of variation (CV), time in tight range (TITR), and time in range (TIR). ICI > 50% indicates improvement on most intervention days. Data shown as mean and 95% CI. The *P* value for each TITR group indicates that the ICI value within that subgroup differs significantly from 50%, evaluated using a two-tailed sign-flip permutation test.

The ICI was evaluated independently within each baseline TITR group to determine whether the group’s mean values differed significantly from 50% (Figure 4). The low TITR group showed significant improvement across multiple metrics: GRI (72.1% [59.9%-83.7%]), TIR (70.5% [58.2-81.8]), and CV (73.3% [63.9-82.2]), all *P* < .01. The moderate TITR group showed significant consistency in TITR (72.5% [62.7-80.9]) and CV (68.8% [59.6-77.6]), both *P* < .001. The high TITR group achieved consistent CV improvement (69.5% [62.6-76.2], *P* < .001). By self-reported baseline diagnosis, T2D individuals showed consistent improvements across all metrics except AG, while the individuals with prediabetes improved only CV, and the metabolically healthy individuals improved both TITR and CV (Supplementary Figure 2).

### Self-reported Engagement and Behavioral Changes

#### Program Engagement

Participants reported frequent engagement (most or all of the time) with program modalities: newsletters (78%), online portals (58%), online videos (57%), and webinars (48%). Overall, 84% frequently engaged with at least one modality (Supplementary Table 1). The median program engagement score was 14 (IQR: 10-17) on a scale from 0 to 20 (Supplementary Figure 3).

#### CGM Engagement

Participants agreed or strongly agreed that CGM had a positive impact on the following behavioral aspects: food choices (94%), exercise choices (73%), stress management (42%), and sleep (39%). Most participants (96%) agreed or strongly agreed that CGM had a positive impact on at least one behavioral aspect (Supplementary Table 1). The median CGM engagement score was 20 (IQR: 17-21) on a scale from 0 to 24 (Supplementary Figure 3).

#### Behavioral Changes

Participants reported the following behavioral changes compared to program start: 78% reported lower carbohydrate intake, followed by reduced sugar intake (71%), eating smaller portions (69%), healthier food consumption (66%), increased physical activity (66%), and less frequent eating out (48.5%). Overall, 98.5% reported at least one positive behavioral change (Supplementary Table 1). The median behavior change score was 4 (IQR: 3-5) on a scale from −6 to 6 (Supplementary Figure 3).

### Pathways Connecting MWP Study to Glycemia Improvements

**Figure 5.**
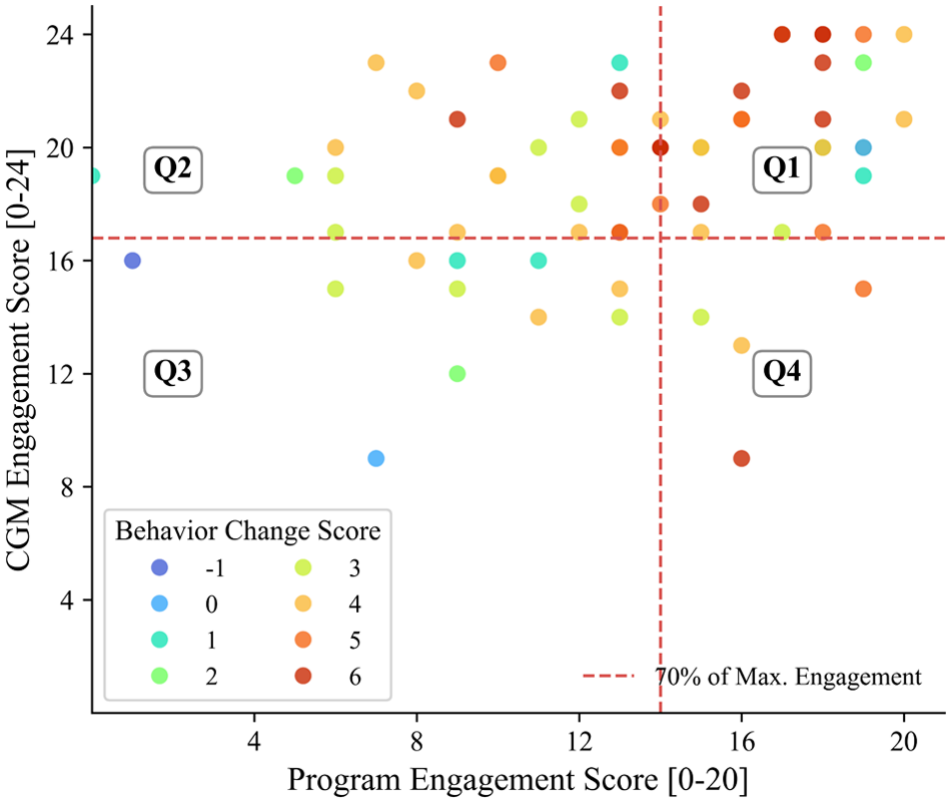
Engagement quadrant stratification. Scatterplot showing program engagement score vs CGM engagement score. Dashed lines at 70% of each axis’s maximum divide participants into four engagement quadrants: Q1 (high program and high CGM engagement), Q2 (high CGM, but low program engagement), Q3 (low program and CGM engagement), and Q4 (high program but low CGM engagement). Each point represents a participant, colored by behavior change score.

**Figure 6.**
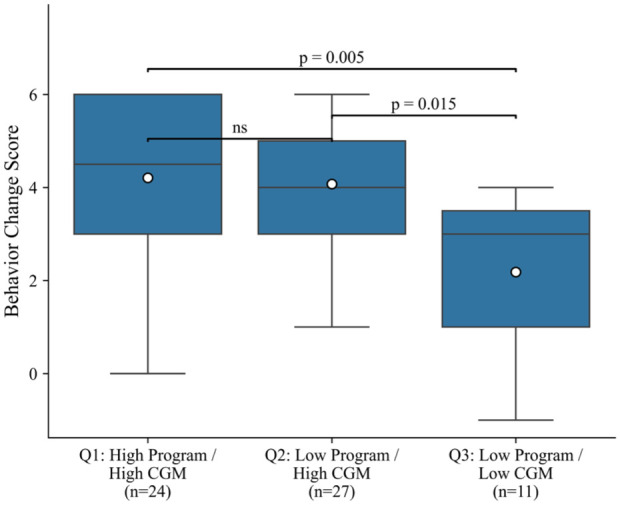
Behavior changes by engagement quadrant. Boxplots represent the median and IQR. White circles represent group means. Group Q3 (median: 3 [IQR: 1.0-3.5]) showed significantly lower behavioral change score than Q1 (median: 4.5 [IQR: 3-6]) and Q2 (median: 4.0 [IQR: 3-5]), both *P* < .05, evaluated using the Kruskal-Wallis test followed by Dunn’s post-hoc test with Bonferroni correction. Q4 was excluded from analysis due to sample size limitation (n = 4).

To evaluate the relationship between engagement and behavioral changes, participants were stratified into four quadrants based on a 70% engagement threshold for both program and CGM engagement scores (Figure 5). Participants with high CGM engagement (Q1 and Q2) achieved significantly higher behavior change scores than participants with low overall engagement (Q3), both *P* < .05 (Figure 6).

### Correlation Between Behavior Changes and Glycemic Outcomes

Using Spearman’s rank correlation, self-reported behavioral changes were positively correlated with TIR MCI (*r* = 0.31 [0.05-0.53], *P* = .02) and GRI MCI (*r* = −0.31[−0.53 to −0.04], *P* = .02), with stronger correlation in the low TITR group for TIR MCI (*r* = 0.57 [0.04-0.85]), TIR ICI (*r* = 0.56 [0.02-0.9]), GRI MCI (*r* = −0.6 [−0.86 to −0.07]), GRI ICI (*r* = 0.64 [0.14-0.88]), AG MCI (*r* = −0.62 [0.87 to −0.11]), AG ICI (*r* = 0.70 [0.2-0.9]), all *P* < .05. Corresponding scatterplots for all absolute correlations greater than 0.6 are provided in Supplementary Figure 4.

### Self-Reported Weight

Participants reported a modest mean weight loss of 4.6 lbs (95% CI: 2.7-6.7; *P* < .001), with greater reductions among those with BMI≥30 (8.6 lbs; 95% CI: 5.2-12.7; *P* < .001).

## Discussion

Lifestyle interventions remain the cornerstone of treatment for T2D and individuals at risk of developing the condition.^18,19^ In this study, health care workforce with diverse glycemic control (normoglycemia to T2D), participated in an 8-week digitally delivered MWP combining education with real-time CGM. Static (pre/post) CGM-derived metrics, such as TIR, showed no apparent glycemic changes associated with program participation. However, applying novel temporal change metrics (MCI and ICI) to AG, GRI, TIR, TITR, and CV captured dynamic glycemic changes throughout the full program duration rather than only at baseline and endpoint. This approach revealed glycemic changes associated with self-reported behavioral changes linked to program engagement, offering opportunities to create personalized bio-behavioral feedback loops.

Applying temporal change metrics revealed significant glycemic changes across baseline strata; the suboptimal glycemic control group (low TITR) improved across all five CGM-derived metrics, while the self-reported T2D group improved across four metrics (excluding AG). These findings contrast with static CGM-derived metrics, which missed these dynamic variations and yielded only an isolated AG improvement within self-reported metabolically healthy individuals. Figure 2 illustrates a participant who improved glycemic control throughout most of the program but experienced worsening control during the final days, coinciding with holidays. Baseline versus endpoint comparisons fail to recognize that participants may improve mid-program, which is then negated by holiday days, when food choices and eating patterns change.^20,21^ Given the known influence of positive messaging on behavior,^22^ detecting these within-program changes offers opportunities for innovative, wearable-based behavioral interventions.

Program and CGM engagement were associated with behavioral changes, which correlated with glycemic improvements. While glycemic control improved, the independent effects of lifestyle education versus CGM cannot be entirely decoupled. Prior research demonstrated that while diabetes education alone improved glycated hemoglobin (HbA1c), adding CGM was associated with greater improvement.^23^ Together, education and CGM components suggest a synergistic loop, where the program educates participants on a healthy lifestyle while CGM provides real-time feedback to reinforce behaviors. The link between CGM use and the application of temporal change metrics to overall program engagement and subsequent positive behavioral changes may create opportunities to build effective bio-behavioral feedback loops and just-in-time adaptive interventions.^24^ These findings support the value of CGM outside the standard of care for insulin therapy.^25,26^

Although weight loss was not a primary aim, participants with paired weight data self-reported an average weight loss of 4.6 lbs, suggesting a modest secondary effect. While subject to self-reporting limitations, this observation aligns with findings where CGM combined with personalized lifestyle recommendations produced comparable weight loss,^27^ and evidence suggesting CGM-supported dietary recommendations without weight loss counseling can reduce weight in individuals with obesity and prediabetes,^28^ though mechanisms remain unclear.

This study has several strengths. The proposed temporal change metrics captured overall progress and day-to-day glycemic changes that static CGM metrics missed, which is critical since glycemic responses to lifestyle interventions are often non-monotonic. Unlike approaches modeling discrete clinical checkpoints,^29^ or complex continuous time-series data,^30,31^ the proposed temporal change metrics serve as intuitive summary metrics capturing the average magnitude and day-to-day consistency of improvement at any given point during intervention. In addition, we examined pathways from engagement to behavioral change to glycemic improvements, revealing significant associations at each step, within a challenging, understudied population of health care workers at increased risk for metabolic syndrome.

Limitations include single-organization results, which may limit generalizability, and the absence of a control group, preventing causal inferences. Baseline diabetes diagnoses, weight, engagement, and behavioral measures were self-reported, introducing recall and social desirability bias. Baseline HbA1c was available for only 41% of participants, restricting definitive stratification or objective verification. Furthermore, paired baseline and post-program HbA1c data were available for only 25% of participants; due to the high risk of attrition bias that could systematically skew results, HbA1c change analysis was not performed. Temporal change metrics also depend on CGM accuracy, meaning atypical baseline periods (eg, illnesses or holidays) could skew true intervention effects. Finally, the modest sample size, lack of blinded baseline CGM, and unknown long-term sustainability warrant caution when interpreting these pilot findings. While these results are preliminary, further studies are planned in subpopulations (eg, in racial and ethnic groups with a disproportionate burden of T2D) with modifications of the core MWP. These future studies will be a randomized controlled clinical trial with appropriate controls.

## Conclusions

Engagement in a digital, CGM-guided lifestyle intervention was associated with behavioral and glycemic improvements within a health care workforce with diverse baseline glycemic control. Novel temporal change metrics detected glycemic improvements that static CGM metrics missed, suggesting their potential to highlight real-time, within-intervention changes that could be used as part of bio-behavioral feedback loops using wearable devices. These findings support the efficacy of digital lifestyle interventions for high-risk workforces, irrespective of baseline glycemic status.

## Supplemental Material

sj-docx-1-dst-10.1177_19322968261463562 – Supplemental material for Temporal Changes in Continuous Glucose Monitoring Profiles in Health Care Workers Participating in a Digitally Delivered Metabolic Wellness Program Supplemental material, sj-docx-1-dst-10.1177_19322968261463562 for Temporal Changes in Continuous Glucose Monitoring Profiles in Health Care Workers Participating in a Digitally Delivered Metabolic Wellness Program by Bariq S. Firmansyah, Ronesh Sinha, Ashutosh Sabharwal, Cheryl D. Stults, Joseph Wilcox, Katie Gillespie, Rekha Viswanathan, Richard Chapman, Susan McClendon and David Kerr in Journal of Diabetes Science and Technology
