# Supplementary material for: Temporal Changes in Continuous Glucose Monitoring Profiles in Health Care Workers Participating in a Digitally Delivered Metabolic Wellness Program
Source: J Diabetes Sci Technol. 2026 Jul 13:19322968261463562. Online ahead of print. doi: 10.1177/19322968261463562 (PMC13368773; doi:10.1177/19322968261463562)
Supplement: sj-docx-1-dst-10.1177_19322968261463562 – Supplemental material for Temporal Changes in Continuous Glucose Monitoring Profiles in Health Care Workers Participating in a Digitally Delivered Metabolic Wellness Program [file sj-docx-1-dst-10.1177_19322968261463562.docx]

**Supplementary Files**

[**Supplementary Table 1.** Survey responses on self-reported engagement with program elements, self-reported engagement with CGM, and self-reported behavior changes at the end of the program compared to the start of the program. 2](#_Toc231294480)

[**Supplementary Figure 1.** Mean Change Index (MCI) of each self-reported diagnosis subgroup (metabolically healthy, pre-diabetes, and type 2 diabetes) in five key metrics: average glucose (AG), Glycemia Risk Index (GRI), coefficient of variation (CV), time-in-tight-range (TITR), and time-in-range (TIR). Positive MCI values indicate improvement for TIR and TITR, while negative MCI values indicate improvement for AG, GRI, and CV. Data shown as mean and 95% CI. The p-value for each subgroup indicates that the MCI value in that subgroup differs significantly from zero, evaluated using a two-tailed sign-flip permutation test. 3](#_Toc231294472)

[**Supplementary Figure 2.** Improvement Consistency Index (ICI) of each self-reported diagnosis subgroup (metabolically healthy, pre-diabetes, and type 2 diabetes) in five key metrics: average glucose (AG), Glycemia Risk Index (GRI), coefficient of variation (CV), time-in-tight-range (TITR), and time-in-range (TIR). An ICI value > 50% indicates improvement on most intervention days. Data shown as mean and 95% CI. The p-value for each subgroup indicates that the ICI value in that subgroup differs significantly from 50%, evaluated using a two-tailed sign-flip permutation test. 4](#_Toc231294473)

[**Supplementary Figure 3.** The distribution of behavior change, program engagement, and CGM engagement scores. 5](#_Toc231294474)

[**Supplementary Figure 4.** Correlation between behavior change score and temporal change metrics. Correlation coefficients (r) and corresponding p-values were evaluated using Spearman’s rank correlation. Scatterplots are presented exclusively for individual relationships with an absolute correlation value greater than 0.6. 5](#_Toc231294475)

| **Survey** | **Responses in % (n/N)** | | | | |
| --- | --- | --- | --- | --- | --- |
| **Self-reported engagement with program elements** | **None of the Time** | **A little of the Time** | **Some of the Time** | **Most of the Time** | **All of the Time** |
| I read the email newsletters | 1·45% (1/69) | 7·25% (5/69) | 13·04% (9/69) | 24·64% (17/69) | 53·62% (37/69) |
| I visited the online resources portal | 5·79% (4/69) | 13·04% (9/69) | 23·19% (16/69) | 28·99% (20/69) | 28·99% (20/69) |
| I watched the videos | 5·79% (4/69) | 13·04% (9/69) | 24·64% (17/69) | 26·09% (18/69) | 30·43% (21/69) |
| I attended the webinars | 18·84% (13/69) | 14·49% (10/69) | 18·84% (13/69) | 37·68% (26/69) | 10·14% (7/69) |
| **Self-reported engagement with CGM** | **Strongly disagree** | **Disagree** | **Neutral** | **Agree** | **Strongly Agree** |
| CGM has a positive impact on my food choice | 0% (0/71) | 0% (0/71) | 5·63% (4/71) | 32·39% (23/71) | 61·97% (44/71) |
| CGM has a positive impact on my exercise choices | 0% (0/71) | 2·82% (2/71) | 23·94% (17/71) | 22·54% (16/71) | 50·70% (36/71) |
| CGM has a positive impact on my sleep | 0% (0/71) | 9·86% (7/71) | 50·70% (36/71) | 18·31% (13/71) | 21·13% (15/71) |
| CGM has a positive impact on how I manage my stress | 0% (0/71) | 4·23% (3/71) | 53·52% (38/71) | 23·94% (17/71) | 18·31% (13/71) |
| **Behavior changes relative to program start** | **Less** | **No changes** | **More** |  |  |
| Meal portion size | 69·12% (47/68) | 29·41% (20/68) | 1·47% (1/68) |  |  |
| Carbohydrate intake | 77·94% (53/68) | 19·12% (13/68) | 2·94% (2/68) |  |  |
| Healthy foods intake | 2·94% (2/68) | 30·88% (21/68) | 66·18% (45/68) |  |  |
| Sugary food intake | 70·59% (48/68) | 25% (17/68) | 4·41% (3/68) |  |  |
| Physical activity level | 0% (0/68) | 33·82% (23/68) | 66·18% (45/68) |  |  |
| Eating out frequency | 48·53% (33/68) | 48·53% (33/68) | 2·94% (2/68) |  |  |

**Supplementary Table 1. Survey responses on self-reported engagement with program elements, self-reported engagement with CGM, and self-reported behavior changes at the end of the program compared to the start of the program.**





**Supplementary Figure 1. Mean Change Index (MCI) of each self-reported diagnosis subgroup (metabolically healthy, pre-diabetes, and type 2 diabetes) in five key metrics: average glucose (AG), Glycemia Risk Index (GRI), coefficient of variation (CV), time-in-tight-range (TITR), and time-in-range (TIR). Positive MCI values indicate improvement for TIR and TITR, while negative MCI values indicate improvement for AG, GRI, and CV. Data shown as mean and 95% CI. The p-value for each subgroup indicates that the MCI value in that subgroup differs significantly from zero, evaluated using a two-tailed sign-flip permutation test.**





**Supplementary Figure 2. Improvement Consistency Index (ICI) of each self-reported diagnosis subgroup (metabolically healthy, pre-diabetes, and type 2 diabetes) in five key metrics: average glucose (AG), Glycemia Risk Index (GRI), coefficient of variation (CV), time-in-tight-range (TITR), and time-in-range (TIR). An ICI value > 50% indicates improvement on most intervention days. Data shown as mean and 95% CI. The p-value for each subgroup indicates that the ICI value in that subgroup differs significantly from 50%, evaluated using a two-tailed sign-flip permutation test.**


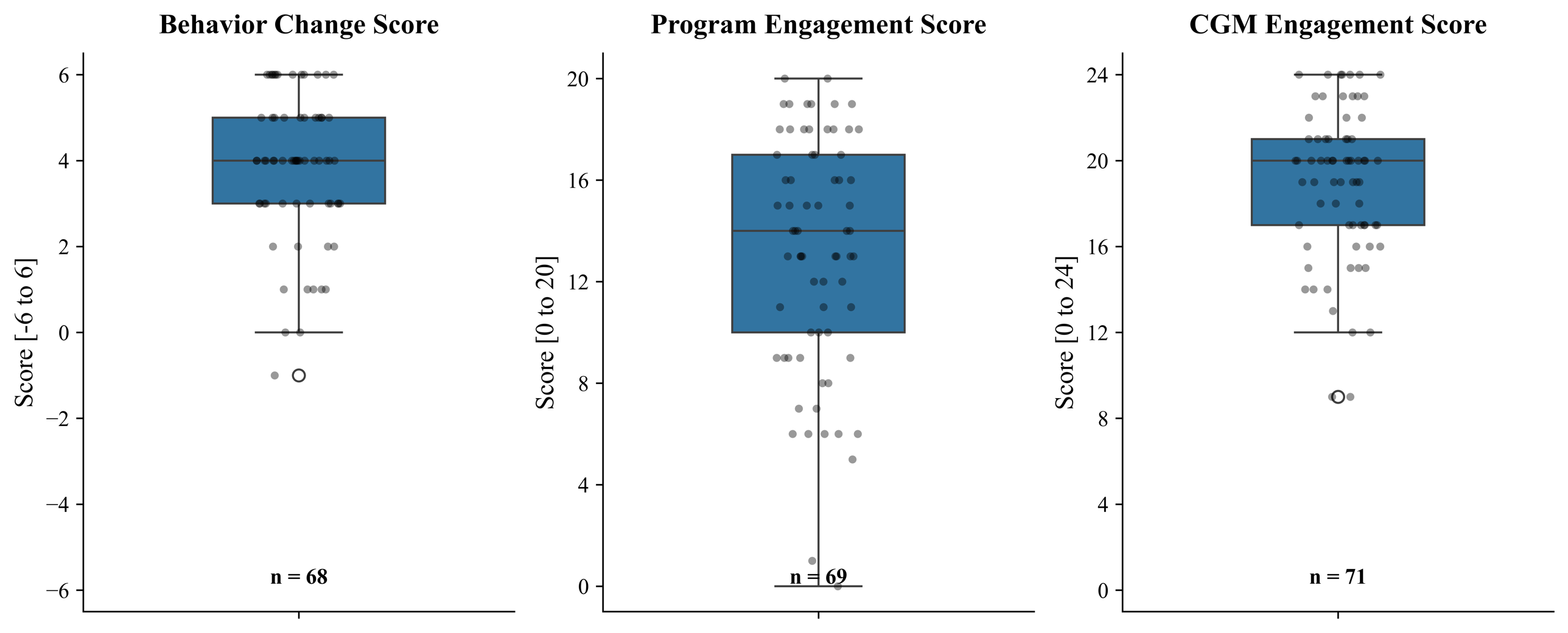


**Supplementary Figure 3. The distribution of behavior change, program engagement, and CGM engagement scores.**


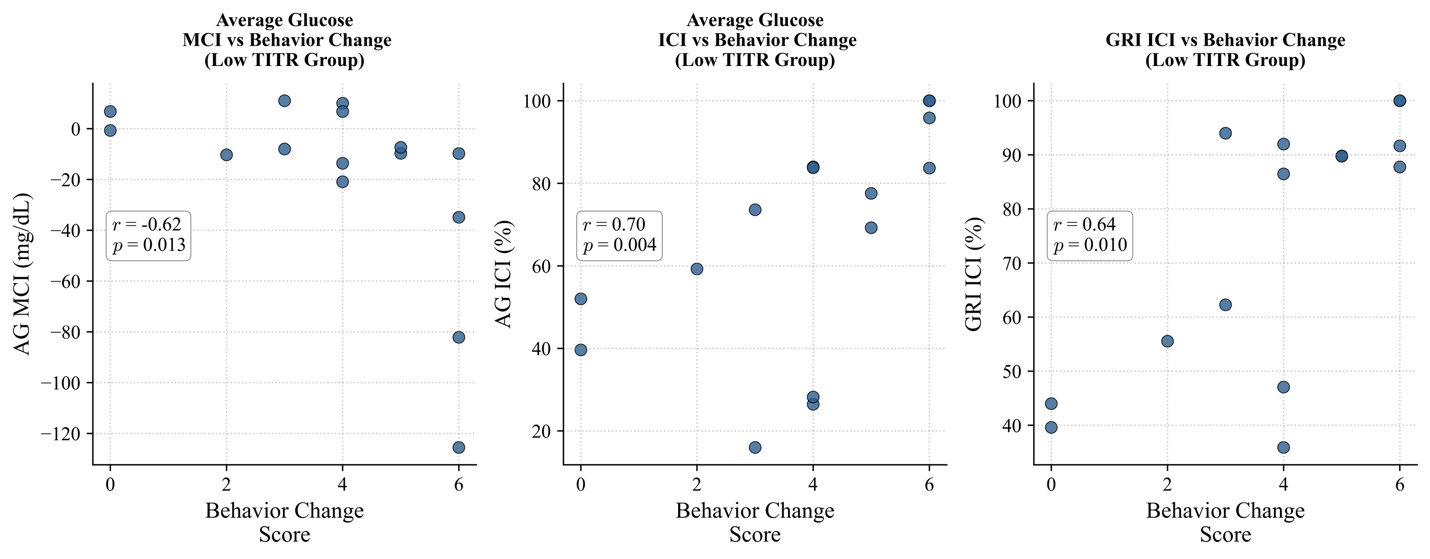


**Supplementary Figure 4. Correlation between behavior change score and temporal change metrics. Correlation coefficients (r) and corresponding p-values were evaluated using Spearman’s rank correlation. Scatterplots are presented exclusively for individual relationships with an absolute correlation value greater than 0.6.**
